# The Quality of Life of Seniors Hospitalized Due to Cardiovascular Diseases in Poland

**DOI:** 10.3390/ijerph17103721

**Published:** 2020-05-25

**Authors:** Katarzyna Sygit, Katarzyna Siedlecka-Pasierbiak, Marian Sygit, Elżbieta Cipora

**Affiliations:** 1 President Stanislaw Wojciechowski State Vocational Academy in Kalisz, Faculty of Health Sciences, 62-800 Kalisz, Poland; msygit@onet.pl; 2 Poddębickie Centrum Zdrowia Hospital, 99-200 Łódź, Poland; katarzyna.pasierbiak@gmail.com; 3 State Vocational Academy in Sanok, Sanok Medical Institute, 38-500 Sanok, Poland; elacipora@interia.pl

**Keywords:** quality of life, the elderly, seniors, health, cardiovascular diseases, physical and psychological well-being

## Abstract

Introduction: In the light of the increased ageing of the world population, social policy needs to be focused on actions aimed at improving the quality of life of older people. Objective: The main objective of this study was to assess the quality of life in a population of seniors hospitalized due to cardiovascular disease. Materials and methods: The study included 408 elderly patients hospitalized for cardiovascular diseases in the Poddębickie Centrum Zdrowia Hospital in Poddębice, Łódzkie voivodship, Poland. The study used two survey questionnaires: the author’s survey questionnaire and the standardized SF36v2 Questionnaire. Statistical analysis of the obtained test results was carried out in the R program, version 3.5.1. Results: Having analyzed the health status of the study group, it was found that the largest group of subjects (84.07%) were treated due to hypertension. Among the ailments that hindered daily functioning, the respondents indicated primarily poor eyesight (53.68%). Patients assessed their own health as ‘mediocre’ (average) (58.58%). The analysis of the study results from the SF36v2 Questionnaire showed that the highest quality of life was in the limited activity due to emotional problems (RE) dimension, social functioning (SF), and physical functioning (PF); the weakest scores were observed in vitality (VT), general health perception (GH), and health transition (HT) dimensions. Conclusions: The significant demographic, social and socio-medical factors that determined respondents’ quality of life were: age, gender, marital status, education and health situation. The analysis of quality of life according to the SF36v2 Questionnaire showed that the study group functioned better in the mental dimension (MCS—mental component summary, overall mental health) than in the physical one (PCS—physical component summary, total physical health).

## 1. Introduction

The forecasts of the United Nations (UN) predict that by 2030, the percentage of Europe’s population aged 65+ will reach 23.8%. Currently, Italy, Germany and Greece are leaders among countries with the highest senior citizen proportion, i.e., people aged 65+ in the general population [1,2,3]. Although in Poland the elderly (65+) constitute over 15% of general population (while the EU average is almost 19%), it should be emphasized that the situation of Poland is only seemingly favorable compared to Europe, because the situation will change radically in the coming decades. By 2050, Poland will become one of the European countries with the highest old age ratio—it will double and amount to over 30%. The oldest age group (80+), which today amounts to about 4% of the general population, also needs to be considered. According to Eurostat, it will reach almost 10% of the general population in 2050 [1]. Research shows that 80+ individuals suffer from an increasing number of diseases, becoming more and more dependent and in need of help [1,4,5,6].

Cardiovascular disease (CVD) is the most frequently mentioned issue among health problems of the elderly; it encompasses ischemic heart disease, stroke, and atherosclerosis, which are the main causes of morbidity, disability and premature mortality in developed countries. The results of the Global Burden of Disease Study indicate that cardiovascular disease has caused about 15.6 million deaths worldwide (29.6%). It is estimated that in Europe, cardiological diseases are the cause of 45% of all deaths, i.e., over 4 million per year [2,3,7,8].

Since 1991, mortality caused by cardiovascular diseases in Poland has systematically decreased; however, it is still considered ‘high’. In 2015, cardiological diseases were the cause of approximately 46.8% of deaths, while they constituted as much as approx. 52% in the early 1990s [1,3,7].

The most pressing problems of the elderly include not only health problems, but also loneliness, disability, poverty, and a sense of uselessness. All these problems point to the existing marginalization of seniors as a group, which may be exemplified by their gradual elimination from active professional and social life [9,10,11].

From the psychosocial perspective, old age is a set of various interrelated social, economic, family and cultural conditions whose impact varies both environmentally and individually; it is a consequence of losses to which the old person is exposed: the loss of social prestige, impoverishment, and excess of free time which is difficult to fill up satisfactorily [3,11].

Old age is a difficult period in human life, and one needs to be properly prepared for it. The development of geriatrics and gerontology results in progress in improving the quality of life in old age. Therefore, extensive actions should be taken to help the elderly with such numerous health, social or economic problems that greatly affect their quality of life [7,10,11].

The quality of seniors’ life has become the subject of numerous studies (especially in recent years) and a topic often raised in scientific articles [12,13,14,15,16,17]. The concept of quality of life is difficult to define, as its perception depends on the worldview, experience and education.

According to the World Health Organization (WHO) definition, quality of life is ‘an individual’s perception of their position in life in the context of the culture and value systems in which they live and in relation to their goals, expectations, standards and concerns’ [9]. The indicators of quality of life include the ability to continue to play one’s life roles, the ability to adapt, psychological well-being and functioning within social groups [9,10].

Undoubtedly, the quality of life changes over time and is significantly susceptible to internal and external factors such as: family life, friendship, work, neighbors, place of residence, home, education, health, standard of living, and nationality [9,10,11]. All these internal and external conditions mean that the quality of life of the elderly is sometimes expressed by:

✓ clinical assessment of physical and mental fitness, susceptibility to diseases, disability;
✓ environmental conditions—housing situation, standard of living, group membership, family status, support systems;
✓ ecological environment—relaxation and recreation facilities in the place of residence, transport conditions;
✓ lifestyle—diet, smoking, drinking alcohol, exercising;
✓ economic and living conditions—income from pension, other forms of financial support [11].

**Figure 1:**
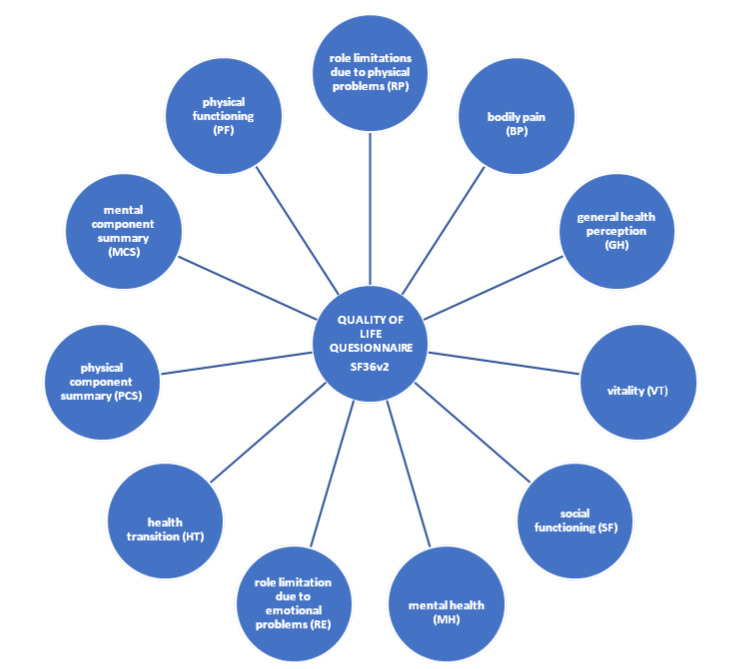
Dimensions used to assess the quality of life in SF36v2 questionnaire.Source: authors’ own work.

Undoubtedly, the quality of life changes over time and is significantly susceptible to internal and external factors. Currently in the developed world, a new lifestyle has emerged: one with a special focus on social life, expanding one’s interests and doing sports. The demand for education and training is on the increase, which is why various forms of activities for seniors (e.g., Third Age Universities) are currently so popular [15,16,18,19,20]. A number of standardized research tools on quality of life have been developed. The most commonly used tool is the SF36v2 Questionnaire, intended for subjective assessment of one’s health, and it was used in this study. The quality of life of the respondents was assessed in 11 dimensions (Figure 1) [21,22,23].

The main objective of this study was to assess the quality of life in a population of seniors hospitalized due to cardiovascular disease.

The main hypothesisis as follows: demographic and social factors, health, as well as social and environmental problems in everyday life influence the quality of life of seniors hospitalized due to cardiovascular disease.

## 2. Materials and Methods 

The study was conducted in 2018 among 408 elderly patients hospitalized for cardiovascular diseases in the Poddębickie Centrum Zdrowia Hospital in Poddębice, Łódzkie voivodship, Poland.

The criteria for the study group were as follows: age 60+; gender women and men diagnosed with cardiovascular disease and hospitalized in the Poddębickie Centrum Zdrowia Hospital Health Centre in Poddębice, admitted to hospital on schedule and based on a referral from a doctor, agreeing to be included in the study. The exclusion criteria were: age below 60, no cardiovascular disease, or no consent for participation in the study.

The method used in the study was a diagnostic survey. The research tools used in the study includedthe authors’ 2-part survey questionnaire. The first part focused on social information, while the second part aimed at investigating health of the study group. The second tool was the SF36v2 Questionnaire, intended for subjective assessment of health. It consisted of 11 questions, containing 36 statements, which helped specify the following elements: physical fitness, limitations due to health condition, pain, general health perception, vitality, social functioning, sense of mental health, limitations caused by emotional problems, change of health condition, physical functioning total physical health, mental functioning total mental health. 

Participation in the study was voluntary and involved completing an anonymous survey questionnaire. The research toolkit was accompanied by the respondent’s statement regarding consent to participate in the study; respondents were given information about the study. The study was conducted by the authors of the paper. Respondents could refrain from completing the questionnaire at any stage. Fromthe moment of agreeing to participate in the study, the collected data about the respondents were stored in a secure place with no access by unauthorized persons. 

Based on the SF36v2 questionnaire (Quality Of Life Questionnaire), the quality of life of the study group in 11 dimensions was assessed: physical functioning (PF); role limitations due to physical problems (RP); bodily pain (BP); general health perception (GH); vitality (VT); social functioning (SF); mental health (MH); role limitation due to emotional problems (RE); health transition (HT); physical component summary (PCS); and mental component summary (MCS).

The quality of life in each dimension was expressed by a number 0–100. Higher numbers indicate a better quality of respondents’ life. There are no standards for SF-36, so it was not possible to assess whether the results achieved by the respondents meant high or low quality of life. However, a comparison of dimensions was made to identify areas with the highest and poorest quality of life.

Questionnaire SF36v2 was made available by the Office of Grants and Scholarly Research and obtained license No. QM046214.

The study was approved by the Bioethics Committee of the Medical University of Lodz, under number RNN/155/18/KE.

The analysis of the obtained test results was carried out in the R program, version 3.5.1.

The analysis of quantitative variables (i.e., expressed in numbers) was performed by calculating the mean, standard deviation, median, quartiles, minimum and maximum values. The analysis of qualitative variables (i.e., not expressed in numbers) was conducted by calculating the number and percentage of occurrences of each value. Comparison of qualitative variables in groups was made using the chi-square test (with Yates correction for 2 × 2 tables) or Fisher’s exact test (only when expected numbers in tables were low). The comparison of the values of quantitative variables in two groups was made using Student’s t test (when the variable had normal distribution in these groups) or the Mann–Whitney test (non-normal distribution). The comparison of the values of quantitative variables in three or more groups was made using ANOVA variance analysis (when the variable had normal distribution in these groups) or the Kruskal–Wallis test (non-normal distribution). After detecting statistically significant differences, posthoc analysis was carried out with Fisher’s Least Significant Difference LSD test (normal distribution) or Dunn’s test (non-normal distribution) to identify statistically significant differences between groups.

Correlations between quantitative variables were analyzed using the Pearson correlation coefficient (when both variables had normal distribution) or Spearman correlation coefficient (otherwise). The strength of correlation was interpreted according to the following scheme:

|r| ≥ 0.9—very strong correlation
0.7 ≤ |r| < 0.9—strong correlation
0.5 ≤ |r| < 0.7—medium correlation
0.3 ≤ |r| < 0.5—weak correlation
|r| < 0,3—very week correlation (negligible) [24].

The normality of variable distribution was tested using the Shapiro–Wilk test.

The analysis adopted the significance level of 0.05. Thus, all *p* values below 0.05 were interpreted as significant correlations.

## 3. Results

### 3.1. The Overview of the Subjects

The group was dominated by women: 227 (55.64%) versus 181 men (44.36%). The age structure of the study group was as follows: the average age was 70.92 (SD = 6.51) and ranged from 64 to 95; the median was 68, therefore half of the group was younger than 68, and the other half was older. In terms of place of residence, the most numerous groups were inhabitants of cities below 100,000—177 people (43.38%), and over 100,000—126 people (30.88%). One hundred and two individuals (25.00%) lived in a rural environment. When analyzing education of the respondents, secondary education (including post-secondary, non-tertiary education) was the most common—reported by 148 people (36.27%), followed by 105 people (25.74%) with vocational education, 102 people (25.00%) with higher education and 43 people (2.45%) with primary education. The structure of marital status of the studied population was as follows: the largest group comprised married people—222 people (54.41%), followed by widows and widowers—92 people (22.55%), the divorced—50 people (12.25 %), single people—26 people (6.37%), while cohabitation partners accounted for 10 people (2.45%). The main source of income for the majority of respondents was disability pension or old-age pension (non-work sources)—263 individuals (64.46%). Other sources of income included work in private institutions and companies—91 people (22.30%), work in budgetary institutions—56 people (13.73%), agriculture—20 people (4.90%), while 7 people (1.72%) were family-dependent. Among other sources of income indicated by the respondents (47 people, 11.52%), the family allowance and rehabilitation allowance were the most common (Table 1).

### 3.2. Health Condition of the Respondents

Analyzing the incidence of treated cardiovascular diseases in the study group, it was found that 343 people (84.07%) had hypertension, followed by varicose veins and venous thrombosis—163 people (39.43%), arrhythmia and cardiac conduction disorders—133 people (32.60%), atherosclerosis—124 people (30.39), coronary heart disease—71 people (17.40%), and heart defects—31 people (7.60%). Respondents also reported other diseases such as stroke and myocardial infarction. Among the ailments that hindered their daily functioning, respondents indicated primarily poor eyesight—219 people (53.68%), hearing impairment—114 people (27.94%), as well as imbalance—90 people (22.06%). Respondents also reported other issues that hindered their everyday life, such as urinary incontinence, headaches, abdominal pains, and chest pains. The results show that the majority of the study group had persistent pain that hampered their everyday life (the most common complaints were back and knee pain)—261 people (63.97%). The majority of the study group—89.46%—took medication prescribed by a doctor. Worryingly, 42 people (10.29%) stated that they did not follow their doctor’s prescriptions. The majority of patients assessed their health as ‘mediocre’ (average)—239 people (58.58%). A group of 91 people (22.30%) assessed their health as ‘good’, 69 people (16.91%) as ‘poor’, and only 2 people (0.49%) as ‘very good’ (Table 2).

### 3.3. Assessment of the Respondents’ Quality of Life with the SF36v2 Questionnaire

The analysis results show that the study group had the best quality of life in the RE, SF and PF dimensions, while it was the lowest in the VT, GH and HT dimensions. The respondents functioned somewhat better in the mental dimension (MCS) than in the physical one (PCS) (Table 3).

There were statistically significant correlations between the quality of life in the PF and PCS dimensions and the gender of respondents (*p* < 0.05). They were significantly higher for men than for women (Table 4).

Analysis of the results found that X correlates significantly and negatively with the quality of life in the PF, RP, BP, VT, SF, RE, PCS and MCS dimensions (since *p* < 0.05); therefore, the older the age of the respondents, the lower the quality of life in these dimensions (Table 5).

The study found that quality of life in all dimensions depended on the education of respondents (*p* < 0.05).

A posthoc analysis was performed, which helped discover that:

- in the BP dimension, respondents with higher education had a higher quality of life than other respondents; and those with secondary education had a higher quality of life than individuals with vocational education;
- in the RP, VT and SF dimensions, respondents with higher education had a higher quality of life than other respondents, and those with secondary education had a higher quality of life than individuals with primary (incomplete) education;
- in the GH and MH dimensions, respondents with higher and secondary education had a higher quality of life than individuals with vocational and primary (incomplete) education;
- in the HT dimension, respondents with higher and secondary education had a higher quality of life than respondents with vocational and primary (incomplete) education, while respondents with vocational education had a higher quality of life than individuals with primary (incomplete) education;
- in the PF, RE, PCS and MCS dimensions, respondents with higher education had a higher quality of life than other respondents; and those with secondary education had a higher quality of life than individuals with vocational and primary (incomplete) education (Table 6).

The results analysis showed that the quality of life in the PF and RE dimensions depended on the marital status of the respondents (*p* < 0.05); it was relatively higher among respondents in relationships (Table 7).

## 4. Discussion

The term ‘quality of life’ is understood as physical and mental well-being, satisfaction with life, happiness, and fulfilment of desires. Its perception is multidimensional and based on the subjective assessment of an individual [15,16,25,26].

The objective of the study was to assess the quality of life in a population of seniors hospitalized due to cardiovascular disease. The research results help formulate a number of valuable conclusions which are presented in the final part of the paper.

Along with the socio-economic development, the increase in the standard of living, the development of production, the appearance of new goods and services, as well as civilizational and cultural changes, the patterns of behavior of the individuals began to change and their life needs increased. Therefore, there is a justified need to conduct research on the quality of life, both from the point of view of the individual and the entire society, which determines the ways, styles and standards of living [12,13,14,27,28,29,30].

The health condition of the respondents was an important element of their quality of life [31,32,33]. Research showed that the majority of respondents (84.07%) were treated for hypertension, while 39.95% were diagnosed with varicose veins and venous thrombosis. 

Nowadays, hypertension is recognized as one of the most pressing health problems in Poland and worldwide [2,7,9,10,11,31,33,34,35,36]. According to a report by the World Health Organization, almost 1 billion people aged 25+ suffer from hypertension. It is a disease with a complex etiology and may be caused by many factors. Hypertension complications are the cause of 12.8% of all deaths worldwide [2,8]. Many studies have observed that in high-income countries, the percentage of people with hypertension is reduced due to the promotion of a healthy lifestyle and access to appropriate therapies [4,5,9,37,38]. In other countries, it is necessary to implement measures to improve the detection and treatment of hypertension [29,37,38,39,40].

Other cardiovascular diseases that are relatively common among seniors include atherosclerosis, coronary heart disease, arrhythmia and heart conduction disorders, and heart defects. In the study group, these were relatively rare, but other studies have shown that atherosclerosis is relatively common in adults; over time, it leads to clinically observable cardiovascular diseases, such as coronary artery disease, stroke, and peripheral atherosclerosis. These diseases are the main cause of death in Poland and around the world [2,3,7,8]. In Europe, cardiovascular diseases are responsible for around 40% of deaths before the age of 75 [2,5,41]. Due to effective prevention and treatment, a significant reduction in mortality due to atherosclerotic diseases has been observed in many European countries [3,5,9,11,36].

The needs and possibilities to access them, as well as associated problems, are an important quality of life factor for people aged 60+ [11,35,36,40].

A significant problem for the respondents was pain, which hindered everyday functioning. This was reported by more than half of the respondents—63.97%. Pain is often found in patients with pre-existing cardiovascular disease [2,5,7,8,34,35]. Pain significantly worsens patients’ quality of life. Numerous studies have yielded interesting results regarding the occurrence of chest pain, lower limbs (oedema of the lower legs), and joint pain [2,3,7,8,35,36,40]. This is a huge problem for patients and their immediate environment.

To improve patients’ health, and thus their quality of life, it is important to take medication prescribed by a doctor. Among the patients, 89.46% indicated that they followed doctor’s instructions regarding medication. However, numerous studies have shown that patients do not take drugs prescribed by their physician due to, e.g., material difficulties, forgetting about it or ignoring medical recommendations [4,6,39,40,42].

When self-assessing their health, 58.58% of respondents declared their health to be mediocre (average), while 22.30% described it as ‘good’. Subjective health self-assessment was important in assessing patients’ quality of life. Studies by other authors have also shown that most patients hospitalized due to cardiovascular disease assess their health worse than ‘mediocre’.

Based on the SF36v2 Questionnaire, the quality of life of the subjects was assessed in 11 dimensions. It was found that the highest quality of life was in the limited activity due to emotional problems(RE) dimension, social functioning (SF), and physical functioning (PF); the weakest scores were observed in vitality (VT), general health perception (GH), and health transition (HT) dimensions. The respondents functioned somewhat better in the mental dimension (MCS) than in the physical one (PCS). In studies on the quality of life with the SF36v2 Questionnaire, other authors came to the conclusion that in the population of people aged 50+, the best quality of life was in the physical fitness (PF) and general health perceptions (GH) dimensions [21,22,23,43,44]. 

The results of this study show that there is a relationship between the quality of life and the gender of the subjects. This relationship was discovered in two dimensions: physical functioning (PF) and PCS (functioning in the physical dimension, total physical health). It was significantly higher in the group of men. In a study conducted by Hopman et al., a similar relationship may be noticed, in particular regarding the highest quality of life in the physical functioning (PF) dimension [23].

The research also confirmed that the quality of life depended on the education of the respondents in each of the dimensions. Similar conclusions were reached by Lam et al. and Wang et al. Education (higher education in particular) and high awareness of health or well-being resulted in a higher quality of life of the respondents [21,22].

The quality of life, as observed in this study, depended on the marital status of respondents. The quality of life was relatively higher among the respondents in relationships. This is confirmed by other studies, which proves that a life in companionship, sharing one’s problems, and support of a loved one in times of illness are priceless and affect both physical and mental health [22,25].

It should be noted that the perception of quality of life by older people is slightly different compared to the rest of the population before the age of 65. When seniors assess their quality of life, they include their physical and mental well-being, satisfaction with life, happiness and fulfilment of desires [15,21,22,45].

Research on the quality of life of the elderly should be a source of information on how to assess patients’ life situation—preferably by means of self-assessment. Such research may provide valuable information on how to proceed with patients [2,3,7,8,32,33,35,46,47].

When assessing seniors’ quality of life, various aspects related to health and physical and mental well-being should be taken into account, as well as social functioning aspects. These reflect a sense of happiness, a vision of oneself, social comparison, coping with changes and limitations, a positive attitude, and determination, which in turn reflect the scope of necessary support [25,26,27,28,29].

## 5. Conclusions

Significant demographic, social and socio-medical factors which determined the quality of subjects’ life included age, gender, marital status, education and health situation, in particular cardiovascular diseases, visual impairment, hearing impairment, balance disorders and backache.The study also showed that most of the respondents assessed their health as ‘average’ and their quality of life was closely correlated with their health.The analysis of quality of life of seniors with pre-existing cardiovascular disease (assessed with the SF36v2 Questionnaire) indicatedthe highest quality of life in terms of physical fitness and social functioning, as well as limited activity caused by emotional problems, andthe lowest quality of life in terms of vitality, perception of one’s own health and health transition. The study group functioned better in the mental dimension (MCS, overall mental health) than in the physical one (PCS, total physical health).

Practical implications: It would be advisable to continue research on the quality of life of seniors in order to discover the social and environmental problems which significantly determine quality of life of the elderly population. Due to the diverse quality of seniors’ lives, depending on gender, age, health, education and marital status, measures should be taken to improve their quality of life, in particular to facilitate the functioning of the elderly in everyday life; additionally, an effective health education implemented in schools from an early age is recommended to reduce socio-medical and financial problems before reaching old age, as these problems may have a significant impact on the quality of life of seniors in the future.

## Figures and Tables

**Table 1:** The overview of the study group.

No.	The Overview of the Study Group		N	%
1.	Gender	Woman	227	55.64
		Man	181	44.36
2.	Age (in years)	Average—70.92	-	-
		SD—6.52		
		Median—68		
		Min—64		
		Max—95		
		Q1—66		
		Q3—74		
3.	Place of residence	City < 100,000 inhabitants	177	43.38
		City > 100,000 inhabitants	126	30.88
		Rural area	102	25.00
		No answer	3	0.74
4.	Education of the subjects	Primary (incomplete)	10	2.45
		Primary	43	10.54
		Vocational	105	25.74
		Secondary, including post-secondary	148	36.27
		Higher. including bachelor’s degree	102	25.00
5.	Marital status	Single	26	6.37
		Married	222	54.41
		In separation	2	0.49
		Divorced	50	12.25
		Widow/widower	92	22.55
		In a partnership	6	1.47
		Cohabitation	10	2.45
6.	Source of income	Work in budgetary institutions	56	13.73
		Work in private institutions and companies	91	22.30
		Agriculture work	20	4.90
		Family-dependent	7	1.72
		Casual work	12	2.94
		Non-profit	263	64.46
		Other	47	11.52

*N*—number of respondents; %—percentage of respondents; SD—average age of respondents.

**Table 2:** Health condition of the respondents.

No.	Health Condition		N	%
1.	Treatment connected to cardiovascular disease (CVD) *	Hypertension	343	84.07
		Atherosclerosis	124	30.39
		Ischemic heart disease	71	17.40
		Rhythm and cardiac conduction disorders	133	32.60
		Heart defects (congenital and acquired)	31	7.60
		Varicose veins. venous thrombosis	163	39.95
		Other	116	28.43
2.	Ailments hindering everyday life *	Eyesight impairment	219	53.68
		Hearing impairment	114	27.94
		Speech disorders	6	1.47
		Speech disorders	90	22.06
		Other	121	29.66
3.	Pain hindering everyday life	Yes	261	63.97
		No	146	35.78
		No answer	1	0.25
4.	Taking medications prescribed by a doctor	Yes	365	89.46
		No	42	10.29
		No answer	1	0.25
5.	Subjective health assessment	Very good	2	0.49
		Good	91	22.30
		Mediocre (average)	239	58.58
		Poor	69	16.91
		Very poor	6	1.47
		No answer	1	0.25

* Percentage does not add up to 100%, as this was a multiple-choice question. *N*—number of respondents; %—percentage of respondents;

**Table 3:** Quality of respondents’ life in individual dimensions.

Dimension	N *	Mean	SD	Median	Min	Max	Q1	Q3
PF	405	56.14	29.82	60	0	100	30	85
RP	408	49.42	25.42	50	0	100	31.25	68.75
BP	407	50.49	20.1	50	10	100	30	70
GH	408	44.47	16.49	45	0	85	35	55
VT	408	46.58	14.75	50	10	90	35	60
SF	408	56.31	23.9	62.5	0	100	37.5	75
RE	408	60.6	27.99	66.67	0	100	50	75
MH	405	52.59	16.13	52	8	88	44	64
HT	408	38.66	20.48	50	0	100	25	50
PCS	404	50.14	19.32	53.03	1.52	90.91	36.36	65.15
MCS	405	52.71	16.05	53.85	6.15	90.77	41.54	66.15

* Some respondents omitted certain questions, thus preventing calculation of some dimensions; PF—physical functioning; RP—role limitations due to physical problems; BP—bodily pain; GH—general health perception; VT—vitality; SF—social functioning; MH—mental health; RE—role limitation due to emotional problems; HT—health transition; PCS—physical component summary; MCS—mental component summary (MCS).

**Table 4:** Quality of life in individual dimensions versus gender of respondents.

Dimension		Women	Men	*p* *
PF	mean ± SD	52.69 ± 29.69	60.44 ± 29.49	0.006
	Median	55	65	NP
	Quartile	30–80	35–90	
RP	mean ± SD	47.47 ± 24.75	51.86 ± 26.1	0.067
	Median	50	50	NP
	Quartile	25–62.5	31.25–68.75	
BP	mean ± SD	49.29 ± 19.83	51.99 ± 20.4	0.198
	Median	50	50	NP
	Quartile	30–60	30–70	
GH	mean ± SD	44.82 ± 16.87	44.03 ± 16.04	0.996
	Median	45	45	NP
	Quartile	35–55	35–55	
VT	mean ± SD	45.51 ± 14.57	47.93 ± 14.92	0.112
	Median	50	50	NP
	Quartile	35–55	40–60	
SF	mean ± SD	54.79 ± 24.44	58.22 ± 23.14	0.159
	Median	50	62.5	NP
	Quartile	37.5–75	50–75	
RE	mean ± SD	58.52 ± 27.54	63.21 ± 28.41	0.117
	Median	58.33	66.67	NP
	Quartile	50–75	50–83.33	
MH	mean ± SD	52.67 ± 15.84	52.49 ± 16.53	0.88
	Median	56	52	NP
	Quartile	44–64	4467	
HT	mean ± SD	38.55 ± 21.71	38.81 ± 18.89	0.826
	Median	50	50	NP
	Quartile	25–50	25–50	
PCS	mean ± SD	48.58 ± 19	52.07 ± 19.6	0.042
	Median	51.52	55.3	NP
	Quartile	34.85–63.64	37.88–67.05	
MCS	mean ± SD	51.81 ± 15.44	53.86 ± 16.77	0.164
	Median	52.31	55.38	NP
	Quartile	40–64.62	43.08–66.15	

* *p* = Normal distribution in groups, Student’s *t*-test; NP = No normal distribution in groups, Mann-Whitney test.

**Table 5:** Quality of life in individual dimensions versus age of respondents.

Dimension	Correlation with Age			
	Correlation Coefficient	*p* *	Correlation Direction	Correlation Strength
PF	−0.356	*p* < 0.001 NP	negative	weak
RP	−0.237	*p* < 0.001 NP	negative	very weak
BP	−0.173	*p* < 0.001 NP	negative	very weak
GH	−0.052	*p* = 0.294 NP	---	---
VT	−0.165	*p* = 0.001 NP	negative	very weak
SF	−0.232	*p* < 0.001 NP	negative	very weak
RE	−0.229	*p* < 0.001 NP	negative	very weak
MH	−0.023	*p* = 0.65 NP	---	---
HT	−0.059	*p* = 0.238 NP	---	---
PCS	−0.289	*p* < 0.001 NP	negative	very weak
MCS	−0.174	*p* < 0.001 NP	negative	very weak

* *p* = normal distribution of both correlated variables, Pearson correlation coefficient; NP = no normal distribution of at least one of the correlated variables, Spearman’s correlation coefficient.

**Table 6:** Quality of respondents’ life in individual dimensions versus their education.

Dimension		(Incomplete) Primary	Vocational	Secondary	Higher	*p* *
PF	Mean ± SD	38.92 ± 30.84	49.33 ± 29.01	58.71 ± 28.59	68.04 ± 26.21	< 0.001
	Median	40	50	65	75	NP
	Quartile	10–65	30–75	35–85	51.25–90	H > S,V,P; S > V,P
RP	Mean ± SD	39.27 ± 23.17	45.42 ± 24.43	48.73 ± 26.79	59.8 ± 22.1	< 0.001
	Median	37.5	50	50	62.5	NP
	Quartile	25–56.25	25–62.5	31.25–68.75	50–75	H > S,V,P; S > P
BP	Mean ± SD	46.98 ± 18.04	43.62 ± 17.33	50.41 ± 20.77	59.51 ± 19.67	< 0.001
	Median	50	50	50	60	NP
	Quartile	30–60	30–50	30–70	50–70	H > S,P,V; S > V
GH	Mean ± SD	38.11 ± 19.91	41.52 ± 16.29	45.71 ± 14.96	49.02 ± 15.46	< 0.001
	Median	40	40	45	50	NP
	Quartile	30–50	30–50	35–55	40–58.75	H,S > V,P
VT	Mean ± SD	40.28 ± 16.85	43.81 ± 14.34	47.03 ± 14.56	52.06 ± 12.32	< 0.001
	Median	40	45	50	55	NP
	Quartile	30–50	30–55	35–56.25	45–60	H > S,V,P; S > P
SF	Mean ± SD	48.35 ± 23	51.67 ± 23.13	56.08 ± 23.21	65.56 ± 23.5	< 0.001
	Median	50	50	62.5	75	NP
	Quartile	25–62.5	37.5–62.5	37.5-75	50–87.5	H > S,V,P; S > P
RE	Mean ± SD	48.27 ± 29.48	55.71 ± 28.62	62.27 ± 27.41	69.61 ± 24.14	< 0.001
	Median	50	50	66.67	75	NP
	Quartile	25–75	41.67–75	50–75	50–83.33	H > S.,V,P; S > V,P
MH	Mean ± SD	43.62 ± 18.7	48.23 ± 15.75	54.66 ± 15.37	58.75 ± 12.72	< 0.001
	Median	44	50	56	60	NP
	Quartile	28–56	36–57	48–64	48–68	H,S > V,P
HT	Mean ± SD	27.36 ± 23.65	35.24 ± 21.29	40.71 ± 19.4	45.1 ± 16.14	< 0.001
	Median	25	25	50	50	NP
	Quartile	0–50	25–50	25–50	50–50	H,S > V,P; V > P
PCS	Mean ± SD	40.05 ± 19.57	45.15 ± 17.8	51.06 ± 19.05	58.99 ± 17.2	< 0.001
	Median	40.91	43.94	54.55	65.15	NP
	Quartile	24.24–56.06	34.85–60.61	36.36–65.15	47.73–71.21	H > S,V,P; S > V,P
MCS	Mean ± SD	44.03 ± 18.26	48.71 ± 14.89	53.94 ± 15.22	59.53 ± 13.98	< 0.001
	Median	44.62	49.23	55.38	61.54	NP
	Quartile	33.85–55.38	36.92–60	44.62–64.62	50.77–69.23	H > S,V,P; S > V,P

* *p* = normal distribution in groups, ANOVA + results of posthoc analysis (Fisher’s LSD test); NP = no normal distribution in groups, Kruskal–Wallis test + posthoc analysis results (Dunn’s test); education: H-higher, S-secondary, V-vocational, P-primary.

**Table 7:** Quality of life in individual dimensions versus marital status of respondents.

Dimension		In a Relationship	Single	*p* *
PF	Mean ± SD	58.86 ± 28.13	52.29 ± 31.74	0.05
	Median	65	55	NP
	Quartile	35–85	25–80	
RP	Mean ± SD	50.29 ± 25.91	48.2 ± 24.73	0.425
	Median	50	50	NP
	Quartile	31.25–68.75	31.25–62.5	
BP	Mean ± SD	50.04 ± 20.53	51.12 ± 19.53	0.43
	Median	50	50	NP
	Quartile	30–70	40–60	
GH	Mean ± SD	44.6 ± 15.36	44.29 ± 17.99	0.659
	Median	45	45	NP
	Quartile	35–55	35–55	
VT	Mean ± SD	47.29 ± 14.95	45.59 ± 14.46	0.292
	Median	50	50	NP
	Quartile	35–60	35–55	
SF	Mean ± SD	56.88 ± 23.71	55.51 ± 24.21	0.785
	Median	62.5	62.5	NP
	Quartile	37.5–75	37.5–75	
RE	Mean ± SD	62.99 ± 29.16	57.25 ± 25.98	0.032
	Median	66.67	58.33	NP
	Quartile	50–89.58	50–75	
MH	Mean ± SD	52.93 ± 16.49	52.12 ± 15.64	0.617
	Median	54	52	NP
	Quartile	44–65	44–64	
HT	Mean ± SD	38.13 ± 20.11	39.41 ± 21.02	0.553
	Median	50	50	NP
	Quartile	25–50	25–50	
PCS	Mean ± SD	51.09 ± 18.51	48.78 ± 20.4	0.407
	Median	53.03	51.52	NP
	Quartile	37.88–65.15	34.85–66.67	
MCS	Mean ± SD	53.6 ± 16.31	51.46 ± 15.65	0.217
	Median	54.62	52.31	NP
	Quartile	42.69–66.15	41.54–64.62	

* *p* = normal distribution in groups, Student’s *t*-test; NP = no normal distribution in groups, Mann–Whitney test.
